# Multicentre validation of the bedside paediatric early warning system score: a severity of illness score to detect evolving critical illness in hospitalised children

**DOI:** 10.1186/cc10337

**Published:** 2011-08-03

**Authors:** Christopher S Parshuram, Heather P Duncan, Ari R Joffe, Catherine A Farrell, Jacques R Lacroix, Kristen L Middaugh, James S Hutchison, David Wensley, Nadeene Blanchard, Joseph Beyene, Patricia C Parkin

**Affiliations:** 1 Department of Critical Care Medicine, Hospital for Sick Children, 555 University Avenue, Toronto, ON, M5G 1X8, Canada; 2 Child Health Evaluative Sciences Program of The Research Institute, Hospital for Sick Children, 555 University Avenue, Toronto, ON, M5G 1X8, Canada; 3 Centre for Safety Research, Hospital for Sick Children, 555 University Avenue, Toronto, ON, M5G 1X8, Canada; 4 Department of Paediatrics, University of Toronto, 555 University Avenue, Toronto, ON, M5G 1X8, Canada; 5 Department of Health Policy, Management and Evaluation, University of Toronto, 155 College Street, Toronto, ON, M5T 3M7, Canada; 6 Institute of Medical Science, 7213 Medical Sciences Building, 1 King's College Circle, University of Toronto, Toronto, ON, M5S 1A8, Canada; 7 Interdepartmental Division of Critical Care Medicine, University of Toronto, 1 Kings College Circle, Toronto, ON, M5S 1A8, Canada; 8 Centre for Patient Safety, University of Toronto, 525 University Avenue, Toronto, ON, M5G 1X8, Canada; 9 Department of Paediatric Intensive Care, Birmingham Children's Hospital, Steelhouse Lane, Birmingham, B4 6NH, UK; 10 Department of Pediatrics, University of Alberta, 8440 112 Street, Edmonton, AB, T6G 2B7, Canada; 11 Department of Pediatrics, Stollery Children's Hospital, 8440 112 Street, Edmonton, AB, T6G 2B7, Canada; 12 Department of Pediatrics, CHU Sainte-Justine, 3175 Chemin de la Côte-Sainte-Catherine, Montréal, QC, H3T 1C5, Canada; 13 Neurosciences and Mental Health Program of The Research Institute, Hospital for Sick Children, 555 University Avenue, Toronto, ON, M5G 1X8, Canada; 14 Department of Paediatrics, University of British Columbia, 4480 Oak Street, Vancouver, BC, V6H 3V4, Canada; 15 Program in Population Genomics, Department of Clinical Epidemiology & Biostatistics, McMaster University, 1280 Main Street West, Hamilton, ON, L8S 4K1, Canada; 16 Dalla Lana School of Public Health, University of Toronto, 155 College Street, Toronto, ON, M5T 3M7, Canada

## Abstract

**Introduction:**

The timely provision of critical care to hospitalised patients at risk for cardiopulmonary arrest is contingent upon identification and referral by frontline providers. Current approaches require improvement. In a single-centre study, we developed the Bedside Paediatric Early Warning System (Bedside PEWS) score to identify patients at risk. The objective of this study was to validate the Bedside PEWS score in a large patient population at multiple hospitals.

**Methods:**

We performed an international, multicentre, case-control study of children admitted to hospital inpatient units with no limitations on care. Case patients had experienced a clinical deterioration event involving either an immediate call to a resuscitation team or urgent admission to a paediatric intensive care unit. Control patients had no events. The scores ranged from 0 to 26 and were assessed in the 24 hours prior to the clinical deterioration event. Score performance was assessed using the area under the receiver operating characteristic (AUCROC) curve by comparison with the retrospective rating of nurses and the temporal progression of scores in case patients.

**Results:**

A total of 2,074 patients were evaluated at 4 participating hospitals. The median (interquartile range) maximum Bedside PEWS scores for the 12 hours ending 1 hour before the clinical deterioration event were 8 (5 to 12) in case patients and 2 (1 to 4) in control patients (*P *< 0.0001). The AUCROC curve (95% confidence interval) was 0.87 (0.85 to 0.89). In case patients, mean scores were 5.3 at 20 to 24 hours and 8.4 at 0 to 4 hours before the event (*P *< 0.0001). The AUCROC curve (95% CI) of the retrospective nurse ratings was 0.83 (0.81 to 0.86). This was significantly lower than that of the Bedside PEWS score (*P *< 0.0001).

**Conclusions:**

The Bedside PEWS score identified children at risk for cardiopulmonary arrest. Scores were elevated and continued to increase in the 24 hours before the clinical deterioration event. Prospective clinical evaluation is needed to determine whether this score will improve the quality of care and patient outcomes.

## Introduction

Timely application of critical care improves patient outcomes [1-4] but depends upon early identification of patients at risk [5,6]. Late recognition resulting in cardiopulmonary arrest occurs in 0.1 to 20 of 1,000 children admitted to hospital inpatient units [7-9] and is associated with poor survival [10] and significant morbidity in survivors [8,11-15].

Systems that distinguish patients at risk for near and actual cardiac arrest from other low-risk, 'well' hospitalised patients will minimise false-alarm calls to critical care teams while identifying patients at risk. To date, few identification systems have undergone methodologically rigorous development and evaluation [6,16-18]. Reviews of adult identification assessments have found high false-positive rates, low sensitivity and modest score performance versus hospital mortality, suggesting that these mechanisms are of limited value [19,20].

We developed the Bedside Paediatric Early Warning System (Bedside PEWS) scoring system using expert opinion and statistical methods. The seven items used to calculate the score are heart rate, systolic blood pressure, capillary refill time, respiratory rate, respiratory effort, transcutaneous oxygen saturation and oxygen therapy (Table 1). The range of possible scores is 0 to 26. In the development data set, in a single-centre study, this seven-item scale was found to have an area under the receiver operating characteristic (AUCROC) curve of 0.91 and a sensitivity of 83% at a score of 8 [6].

**Table 1:** The Bedside Paediatric Early Warning System score items

		Item subscore			
		
Item	Age group	0	1	2	4
Heart rate (bpm)	0 to < 3 months	> 110 and < 150	≥ 150 or ≤ 110	≥ 180 or ≤ 90	≥ 190 or ≤ 80
					
	3 to < 12 months	> 100 and < 150	≥ 150 or ≤ 100	≥ 170 or ≤ 80	≥ 180 or ≤ 70
					
	1-4 years	> 90 and < 120	≥ 120 or ≤ 90	≥ 150 or ≤ 70	≥ 170 or ≤ 60
					
	> 4-12 years	> 70 and < 110	≥ 110 or ≤ 70	≥ 130 or ≤ 60	≥ 150 or ≤ 50
					
	> 12 years	> 60 and < 100	≥ 100 or ≤ 60	≥ 120 or ≤ 50	≥ 140 or ≤ 40
Systolic blood pressure (mmHg)	0 to < 3 months	> 60 and < 80	≥ 80 or ≤ 60	≥ 100 or ≤ 50	≥ 130 or ≤ 45
	3 to < 12 months	> 80 and < 100	≥ 100 or ≤ 80	≥ 120 or ≤ 70	≥ 150 or ≤ 60
	1 to 4 years	> 90 and < 110	≥ 110 or ≤ 90	≥ 125 or ≤ 75	≥ 160 or ≤ 65
	> 4 to 12 years	> 90 and < 120	≥ 120 or ≤ 90	≥ 140 or ≤ 80	≥ 170 or ≤ 70
	> 80 and < 100	> 100 and < 130	≥ 130 or ≤ 100	≥ 150 or ≤ 85	≥ 190 or ≤ 75
Capillary refill time		< 3 seconds			≥ 3 seconds
Respiratory rate (breaths/minute)	0 to < 3 months	> 29 and < 61	≥ 61 or ≤ 29	≥ 81 or ≤ 19	≥ 91 or ≤ 15
	3 to < 12 months	> 24 or < 51	≥ 51 or ≤ 24	≥ 71 or ≤ 19	≥ 81 or ≤ 15
	1 to 4 years	> 19 or < 41	≥ 41 or ≤ 19	≥ 61 or ≤ 15	≥ 71 or ≤ 12
	> 4 to 12 years	> 19 or < 31	≥ 31 or ≤ 19	≥ 41 or ≤ 14	≥ 51 or ≤ 10
	> 12 years	> 11 or < 17	≥ 17 or ≤ 11	≥ 23 or ≤ 10	≥ 30 or ≤ 9
Respiratory effort		Normal	Mild increase	Moderate increase	Severe increase/any apnoea
Oxygen saturation (%)		> 94	91 to 94	≤ 90	
Oxygen therapy		Room air		Any to < 4 L/minute or < 50%	≥ 4 L/minute or ≥ 50%

The objective of this study was to evaluate the performance of the Bedside PEWS score in a larger multicentre study before clinical implementation. We hypothesised that the Bedside PEWS score (1) could identify children at risk for cardiopulmonary arrest with at least one hour's notice, (2) might increase during the time leading up to the clinical deterioration event, (3) would be independent of the number of static risk factors for cardiac arrest and (4) would be superior to the retrospective ratings of nurses.

## Materials and methods

A 1:2 frequency-matched case-control study was performed. The primary outcome was the Bedside PEWS score. Eligible patients were cared for on an inpatient unit other than an ICU and were 18 years of age or younger at the time of hospital admission. Case patients were defined as those who experienced a clinical deterioration event resulting in either an immediate call to the resuscitation team or an urgent ICU admission without a resuscitation team call. An urgent ICU admission was defined as an admission to an ICU in an unscheduled fashion. ICU admission episodes either following a scheduled procedure, directly from an emergency department or from outside the hospital were excluded. Control patients were defined as those who were cared for on an inpatient unit without resuscitation team call or urgent ICU admission during the period studied or for the following 48 hours.

The children were not studied while they were in an ICU, emergency department or operating room or if they were in the care of an anaesthetist for procedural sedation in another area. We excluded children for whom care was either undergoing or anticipated to undergo medicolegal review as well as those with care restrictions.

### Matching

Matching was performed as follows. First, clusters of similar types of inpatient units were identified within each hospital. For example, all the general surgical units comprised one cluster, and another cluster was composed of the units caring for bone marrow transplant recipients and oncology patients. Patients within each cluster who were in the same Bedside PEWS age category (< 3 months, 3 months to < 12 months, 1 year to < 5 years, 5 to 12 years and > 12 years) were frequency-matched at a ratio of two control patients per case patient.

Clinical data, including 14 risk factors for cardiopulmonary arrest, were obtained by direct abstraction from medical records using standardised data collection forms (Table 2). Consenting nurses were interviewed to provide additional clinical data that was observed but not documented, and they completed a survey to describe their retrospective global rating of the risk of a clinical deterioration event. They were asked, 'How surprised would you be if your patient had a patient emergency while you were on break'? Responses were recorded on a five-point Likert scale (Table 3).

**Table 2:** Performance of the Bedside Paediatric Early Warning System scores in 2,074 hospitalised children^a^

	Case patients		Control patients			
			
Patient characteristics	*n*	Median score (IQR)	*n*	Median score (IQR)	*P *value	AUCROC (95% CI)
All	686	8 (5 to 12)	1,388	2 (1 to 4)	< 0.0001	0.87 (0.85 to 0.89)
Urgent ICU	381	10 (7 to 13)	772	2 (1 to 4)	< 0.0001	0.92 (0.90 to 0.94)
Code blue	305	6 (3 to 10)	616	2 (1 to 4)	< 0.0001	0.81 (0.78 to 0.84)
Age						
< 3 months	190	7 (4 to 10)	333	2 (1 to 4)	< 0.0001	0.83 (0.79 to 0.86)
3 to < 12 months	164	8 (6 to 11)	362	2 (1 to 4)	< 0.0001	0.86 (0.82 to 0.90)
1 to < 5 years	134	9 (5 to 13)	286	2 (1 to 4)	< 0.0001	0.90 (0.87 to 0.93)
5 to 12 years	110	10 (5 to 13)	221	2 (1 to 3)	< 0.0001	0.89 (0.84 to 0.93)
> 12 years	88	11 (6 to 14)	186	3 (2 to 4)	< 0.0001	0.91 (0.87 to 0.95)
Hospital						
A	324	9 (6 to 12)	658	2 (1 to 4)	< 0.0001	0.88 (0.85 to 0.90)
B	238	6 (4 to 9)	478	1 (1 to 3)	< 0.0001	0.89 (0.86 to 0.92)
C	80	12 (9 to 15.5)	164	5 (2 to 6)	< 0.0001	0.91 (0.87 to 0.95)
D	44	9 (4 to 12)	88	2 (1 to 3)	< 0.0001	0.89 (0.83 to 0.96)
Chronic disease						
Transplant	58	11 (7 to 12)	73	2 (1 to 3)	< 0.0001	0.94 (0.90 to 0.98)
Heart disease	233	9 (6 to 11)	386	3 (2 to 5)	< 0.0001	0.85 (0.81 to 0.88)
Severe cerebral palsy	62	10 (7 to 13)	34	2 (1 to 4)	< 0.0001	0.92 (0.86 to 0.98)
Medical device						
Tracheostomy	57	7 (4 to 11)	36	4 (1.5 to 5.5)	0.0002	0.75 (0.67 to 0.86)
Feeding tube	138	10 (6 to 13)	112	3 (1 to 5)	< 0.0001	0.87 (0.82 to 0.91)
Home oxygen	47	9 (6 to 11)	27	5 (2 to 7)	0.0001	0.81 (0.69 to 0.90)
Acute condition						
Seizures > 15 minutes	47	6 (3 to 10)	6	2 (2 to 4)	0.133	0.73 (0.48 to 0.99)
DKA	0	NA	3	2 (1 to 2)	NA	NA
Complexity						
> 3 services	164	9 (6 to 12)	136	3 (1 to 5)	< 0.0001	0.87 (0.83 to 0.91)
> 10 medications/day	162	10 (6 to 13)	109	3 (2 to 5)	< 0.0001	0.85 (0.81 to 0.90)
Recent transition						
Recent primary service transfer	18	7 (3 to 8)	5	1 (0 to 2)	0.048	0.89 (0.75 to 1.00)
Previous ICU admission	345	8 (6 to 11)	386	3 (1 to 5)	< 0.0001	0.84 (0.81 to 0.87)
< 24 hours after surgery	55	7 (4 to 10)	61	2 (1 to 4)	< 0.0001	0.79 (0.71 to 0.88)

^a^IQR = interquartile range; AUCROC = area under the receiver operating characteristic curve; DKA = diabetic ketoacidosis; 95% CI: 95% confidence interval; NA: not applicable. Data are from 2,074 patients admitted to four university-affiliated paediatric hospitals. Scores were calculated from 23,288 hours with one or more items of the seven-item Bedside PEWS score.

**Table 3:** The maximum Bedside Paediatric Early Warning System scores by retrospective nurse rating of surprise 'if your patient had a patient care emergency while you were on break'^a^

Nurses' rating	All patients		Case patients		Control patients		
**Degree of surprise**	***n***	Maximum BPEWS score*	***n***	**Maximum BPEWS score^b^**	***n***	**Maximum BPEWS score^b^**	***P *value**
Not at all (1)	99	10 (7 to 14)	94	10.5(7 to 14)	5	2 (1 to 9)	0.020
Not very (2)	179	8 (5 to 12)	139	10 (6 to 13)	40	4 (1.5 to 6)	< 0.0001
Somewhat (3)	203	5 (2 to 9)	85	9 (5 to 12)	118	3 (2 to 5)	< 0.0.001
Very (4)	601	2 (1 to 4)	89	6 (3 to 9)	512	2 (1 to 3)	< 0.0001
Extremely (5)	395	2 (1 to 3)	31	5 (3 to 8)	364	2 (1 to 3)	< 0.0001
All	1,477	3 (1 to 6)	438	9 (5 to 12)	1,039	2 (1 to 3)	< 0.0001

^a^Data are from 1,477 children in admitted to inpatient wards of four university-affiliated hospitals. Bedside PEWS (BPEWS) scores were higher in case patients compared with all control patients in each surprise category. ^b^Median (IQR) score.

Clinical data were abstracted by trained research nurses. The clinical data and age required to calculate the Bedside PEWS score were written into case report forms and entered into a custom-made Oracle database (Oracle Corp., Redwood Shores, CA, USA). Entered data were electronically checked for internal consistency of dates and manually rechecked for accuracy. Inconsistencies were resolved by reviewing case report forms and medical records as required.

### Analysis

Clinical data were grouped into 1-hour blocks for 24 hours ending at the event for case patients or at the end of 12 hours of data collection for control patients. Where there were missing data, the most recent recorded data were used, consistent with the approach used for other scores in critically ill children [21]. The greatest subscore for each item within each hour was identified and used to calculate the Bedside PEWS score for that hour. We then calculated the maximum PEWS score for the 12 hours ending 1 hour before the clinical deterioration event and in the six 4-hour blocks preceding ICU admission in patients urgently admitted to the ICU.

The primary analysis evaluated the hypothesis that the Bedside PEWS score can identify children at risk for cardiopulmonary arrest with at least one hour's notice. Logistic regression was used to compare the maximum Bedside PEWS score of case and control patients using all 12 hours of data in control patients and the 12 hours of data ending 1 hour before either urgent ICU admission or a code blue event in the case patients. The AUCROC curve was determined from the c-statistic calculated by logistic regression, and the 95% confidence interval (95% CI) was calculated using an accepted algorithm [22]. The ROC curve was represented graphically, and the sensitivity and specificity of the score at thresholds of 7 and 8 were calculated based on our previous work [6].

Repeated measures linear regression was used to evaluate the temporal evolution of scores preceding urgent ICU admission and code blue events in case patients. The dependent variable was the maximum Bedside PEWS score for each of the six 4-hour time periods preceding the clinical deterioration event. The independent variable was the midpoint of the time interval expressed in hours from the time of ICU admission. Linear regression was used to evaluate the relationships between the maximum Bedside PEWS score and the number of risk factors for cardiac arrest. Separate analyses were performed for case and control patients.

The association between the retrospective rating of nurses and the case or control status of patients was evaluated using logistic regression. We used clinical data from the 12 hours ending 1 hour before the clinical deterioration event and for 12 hours in control patients to calculate the maximum Bedside PEWS score. These data were paired with corresponding survey data from frontline nurses. When more than one nurse was surveyed in this time period, we used the data from the nurse who had last cared for the patient. The responses of the frontline nurses were represented on a numerical scale from 1 to 5. We tabulated the maximum Bedside PEWS score for each level of nurse rating in case and control patients. Logistic regression was used to evaluate the performance of nurse rating, the Bedside PEWS score, and the nurse rating with the Bedside PEWS score. We used the c-statistic as a measure of the AUCROC curve and calculated the 95% CI. Comparison of the AUCROC curve for the nurse rating and the maximum Bedside PEWS score was carried out as described by DeLong *et al*. [23].

Subgroup analyses described score performance in the following patient categories: urgent ICU patients, code blue patients, those who fell within any of the five age categories of the Bedside PEWS score, across institutions, patients with chronic conditions (bone marrow or organ transplantation, cardiac disease, severe cerebral palsy), patients with medical devices that might have place them at increased risk (tracheostomy, enterostomy feeding device, home oxygen), patients with acute illness (diabetic ketoacidosis, seizures), patients whose conditions had increased complexity (> 3 services involved in care, > 10 medications), patients with an administrative risk (recent transfer of primary service, ICU transfer, postoperative, off-service patient), and patients who had cardiopulmonary arrest. Power calculations based on our previous work suggested that differences between means could be shown with 30 patients per group. Given that our objectives were to evaluate score performance within specified subgroups and at each hospital, we sought to maximise the numbers of cases and controls from participating hospitals. Numbers were thus determined by the duration of the study at each hospital. The protocol was reviewed and approved by the research ethics boards at participating hospitals. All research ethics boards required consent for staff participation in the surveys and waived the need for patient consent.

## Results

The study was conducted from August 2004 to January 2009 over 120 hospital months in the 4 participating hospitals (Table 4). The 2,074 patients studied ranged in age from 0 to 227 months, had a median (interquartile range (IQR)) age of 12 months (3.5 to 74) and comprised 686 case patients and 1,388 control patients. There were 305 code blue cases and 381 patients who were urgently admitted to the paediatric intensive care unit without a code blue event (Table 2). There were 23,288 hours with data describing one or more of the Bedside PEWS score items, 7,263 hours (31.2%) when 5 or more of the items of the Bedside PEWS score were used for score calculation, and 1,181 hours (5.1%) when all 7 items were used for score calculation.

**Table 4:** Dates of patient data from each hospital^a^

Hospital	First patient	Last patient
Hospital for Sick Children, Toronto, ON, Canada	16 March 2004	17 March 2008
Birmingham Children's Hospital, Birmingham, UK	21 August 2004	17 October 2006
Stollery Children's Hospital, Edmonton, AB, Canada	14 March 2006	26 April 2008
Saint Justine Hospital, Montreal, QC, Canada	3 April 2007	7 January 2009

^a^Dates given are the earliest and latest dates that patient data were obtained at each hospital.

**Figure 1:**
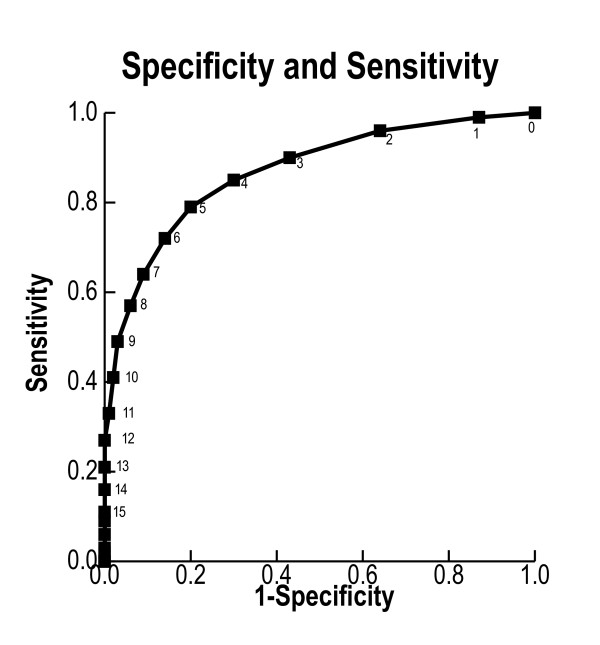
**The receiver operating characteristic curve for the performance of the Bedside Paediatric Early Warning System score**. Data are presented for 2,074 patients who were admitted to inpatient wards of four university-affiliated paediatric hospitals in a frequency-matched case-control study with two control patients per case. Case patients had either an immediate call to a resuscitation team or were urgently admitted to a paediatric intensive care unit (PICU) without a call to the resuscitation team. Control patients had neither. The maximum Bedside Paediatric Early Warning System score was calculated for the 12 hours ending 1 hour before the resuscitation team call or urgent PICU admission in case patients and for 12 hours in control patients.

The median (IQR) of the maximum Bedside PEWS scores was higher in case patients (8 (5 to 12)) than in the 1,387 controls (2 (1 to 4); *P *< 0.0001) (Table 5). The AUCROC curve was 0.87 (95% CI 0.85 to 0.89). When we used a threshold score of 7, the sensitivity was 0.64 and the specificity was 0.91. With a threshold score of 8, the sensitivity was 0.57 and the specificity was 0.94 (Figure 1). Within age, disease and comorbidity subgroups, and within each of the four hospitals, the Bedside PEWS score was able to discriminate case patients from control patients (Table 2).

**Table 5:** Scores and outcomes of case and control patients^a^

Score	Case patients, *n *(%)	Control patients, *n *(%)
0	7 (1.02%)	175 (12.61%)
1	21 (3.06%)	324 (23.34%)
2	36 (5.25%)	294 (21.11%)
3	38 (5.54%)	185 (13.33%)
4	42 (6.12%)	131 (9.44%)
5	44 (6.41%)	86 (6.20%)
6	57 (8.31%)	75 (5.40%)
7	47 (6.85%)	44 (3.17%)
8	47 (6.85%)	28 (2.02%)
9	55 (8.02%)	21 (1.51%)
10	56 (8.16%)	10 (0.72%)
11	44 (6.41%)	5 (0.36%)
12	43 (6.27%)	8 (0.58%)
13	36 (5.25%)	0 (0%)
14	31 (4.52%)	0 (0%)
15	21 (3.06%)	1 (0.07%)
16	22 (3.21%)	2 (0.14%)
17	16 (2.33%)	0 (0%)
18	11 (1.60%)	0 (0%)
19	4 (0.58%)	0 (0%)
20	0 (0%)	0 (0%)
21	1 (0.15%)	0 (0%)
22	1 (0.15%)	0 (0%)
23	1 (0.15%)	0 (0%)
24	1 (0.15%)	0 (0%)
25	0 (0%)	0 (0%)
26	0 (0%)	0 (0%)

^a^Maximum Bedside Paediatric Early Warning System scores for the 12 hours ending one hour before the event in case patients and for 12 hours of data in control patients.

**Figure 2:**
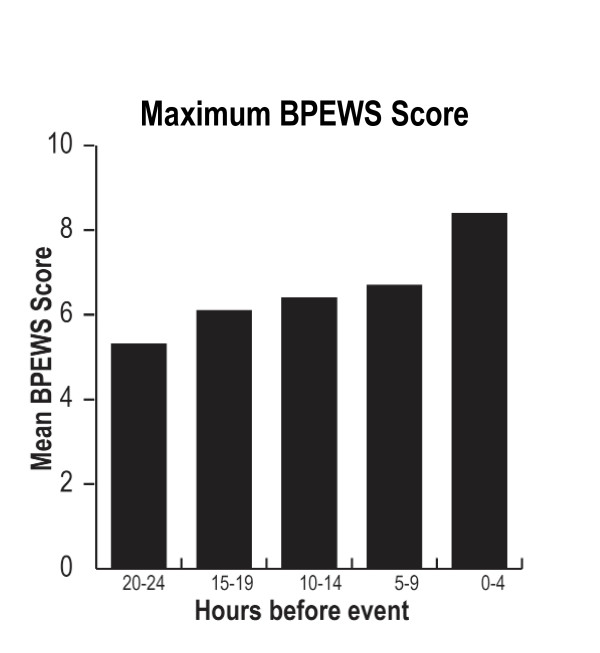
**Progression of Bedside Paediatric Early Warning System scores over time preceding clinically relevant events signifying clinical deterioration**. Data are from 686 patients in the 24 hours before their event: either a call for immediate assistance from a resuscitation team or urgent admission to the paediatric intensive care unit. The graph represents the mean value of the maximum Bedside Paediatric Early Warning System (BPEWS) score from each of the studied patients for the defined four-hour periods. Repeated measures regression shows that the scores increased as the event grew nearer (*P *< 0.0001).

Repeated measures analysis showed that the Bedside PEWS scores increased over the 24 hours before urgent ICU admission or code blue event from a baseline mean Bedside PEWS score of 5.3, 20 to 24 hours before clinical deterioration, to 8.4 in the last 4 hours ending at the code blue event or urgent ICU admission (Figure 2). For each hour closer to the event, the maximum Bedside PEWS score was 0.13 units higher (*P *< 0.0001). When data from the hour immediately before the event were included, the AUCROC curve increased to 0.88 (0.87 to 0.90).

**Figure 3:**
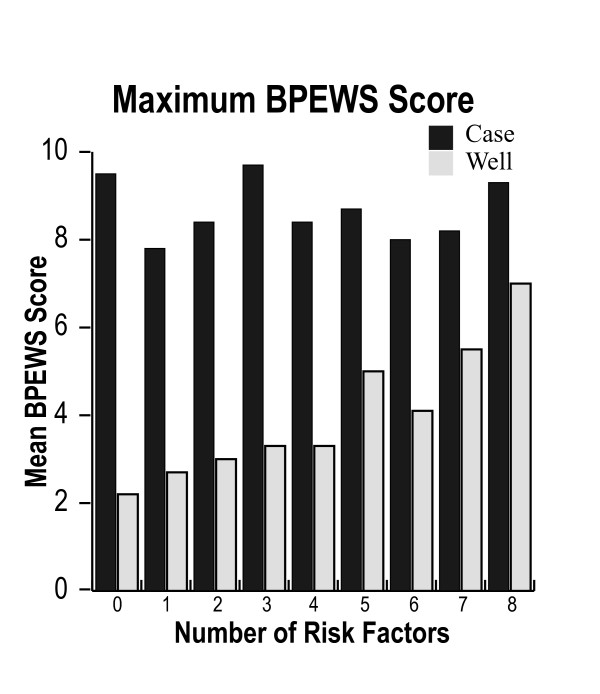
**The relationship between the number of risk factors for near and actual cardiopulmonary arrest and the maximum Bedside Paediatric Early Warning System (BPEWS) score for the 12 hours ending 1 hour before immediate call to a resuscitation team or urgent paediatric intensive care unit admission in 686 case patients and for 12 hours in 1,388 control patients with no events**. The maximum BPEWS score was not related to the number of risk factors in case patients and was positively associated in control patients (*P *< 0.0001).

One or more risk factors were present in 1,698 patients (81.4%), with a median (IQR) of 2 (1 to 4) risk factors in case patients and 1 (0 to 2) in control patients. The number of risk factors was not associated with the maximum Bedside PEWS score (*P *= 0.85) in the 686 case patients. However, in the 1,388 control patients, an increasing number of risk factors were significantly associated with the maximum Bedside PEWS score (*P *< 0.0001). For every additional risk factor, the predicted value of the maximum Bedside PEWS score was 0.327 Bedside PEWS score points higher (Figure 3).

There were 1,477 patients (71.2%) with retrospective nurse ratings describing the 12 hours before the clinical event. When we evaluated these 438 case patients (63.8%) and 1,039 control patients (74.8%) using logistic regression, we found that retrospective nurse ratings were able to discriminate case from control patients (*P *< 0.0001) and that, within the strata of nurse ratings, the Bedside PEWS score was higher in case patients than in control patients (Table 3). The AUCROC curve (95% CI) for the retrospective nurse ratings was 0.83 (0.81 to 0.86). This statistic was significantly lower (*P *< 0.0001) than that for the maximum Bedside PEWS score alone, which was 0.89 (0.88 to 0.91), and was also significantly lower (*P *< 0.0001) than the combination of the maximum Bedside PEWS score and the retrospective nurse ratings combined, which was 0.92 (0.90 to 0.94).

## Discussion

We conducted a prospective multicentre validation of the Bedside PEWS score using a frequency-matched case-control design. In our study of 2,074 patients at 4 university-affiliated centres, we found that the Bedside PEWS score was able to identify patients at risk with at least one hour's notice. Scores were significantly higher in children who had either an urgent ICU admission or a code blue event than in hospitalised children without events (8 versus 2; *P *< 0.0001), increased during the time leading up to clinical deterioration events, and were independent of the number of risk factors for cardiac arrest in case patients. The AUCROC curve was 0.87 (0.85 to 0.89), with scores maintained across age groups, diagnoses and hospitals (Table 2). Three of twenty subgroups evaluated had an AUCROC curve < 0.8. All had wide confidence intervals.

The calculated score preceding the event was superior to the retrospective rating of the frontline nurses who were providing care to the patients who were scored. A greater difference might have been found if the ratings of nurses blinded to patient outcomes had been used. We were able to calculate the score using all 7 items during 1,181 hours (5.1%). It is likely that integration of the score into clinical activities will improve documentation and score performance [24].

Our data suggest that the Bedside PEWS score has the potential to improve existing systems of care by facilitating the timely identification of children at risk for cardiopulmonary arrest. The reported sensitivity and specificity data are likely to improve if implementation of the Bedside PEWS score is associated with the introduction of recommendations for the time of documentation and observation linked to the score. For example, in a hospital with 10 urgent ICU admissions and code blue events per 1,000 patient days, our data suggest that with a threshold score of 7 there will be 59 false-positives and 6 true-positives. This corresponds to a positive predictive value of 9%. This rate seems reasonable, given that cardiopulmonary arrest in patients cared for in hospital inpatient units is associated with high mortality and acquired neurocognitive injury [8,12-15], as is urgent admission to the ICU from inpatient units among patients without cardiopulmonary arrest [25]. Importantly, patients who had low scores in association with events may be systematically different from other patients. Improved understanding this subgroup is likely to improve predictive accuracy.

### Comparison with other studies

The three other published paediatric scores are from studies conducted in Brighton, UK [26]; Toronto, ON, Canada [6,17]; and Cardiff, UK [27]. There are no published multicentre data describing the performance of these scores, and validation studies have reported small numbers of patients with adverse outcomes. The validation studies included 51 urgent ICU admissions [28], 16 patients with 'code blue' events plus 170 rapid response team consultations for the Brighton score [29], 16 clinical deterioration events (death, urgent ICU admission) for the Cardiff score [27] and 87 patients with immediate calls to the resuscitation team for the Toronto score [17]. The current study included 686 patients with adverse events.

We found that a Bedside PEWS score of 7 or higher correctly identified 1,263 of 1,388 control patients. This specificity of 91% compares favourably with the 93% specificity found in the initial validation of the Bedside PEWS score [6], the 95% specificity of the Toronto score [17], the 90% specificity of the Cardiff score [27] and the 82% specificity of the Brighton score [28].

In this study, we found that a Bedside PEWS score of 7 identified 439 of 686 case patients with at least one hour's notice. This sensitivity of 64% is less than the 82% sensitivity reported in the initial validation of the Bedside PEWS score [6], less than the 85.5% sensitivity when a retrospective study design was used for the Brighton score [29], and similar to the initial validation studies reporting sensitivities of 70% for the Cardiff score [27] and 71% for the Brighton score [28].

Despite these similarities, there are several important differences between our study and the previous validation studies [27-29]. First, the Brighton and Cardiff score validation studies included data until the time of event, thus increasing the apparent performance of these scores [27-29]. Both the Toronto score and the Bedside PEWS validation studies used data ending one hour before the event. This approach was used to ensure that hospital staff had sufficient time to respond to the elevated score and to exclude measurement of data documented during a cardiac or respiratory arrest. Second, in our study, the score items and the calculated score were not available to the treating team and thus could not influence decisions. In both the Brighton and Cardiff scores, the documentation charts were modified to better capture the score items and consequently might have influenced treatment decisions (perhaps appropriately), thus increasing apparent score performance [27,28]. Third, in the Brighton score validation studies, charge nurses retrospectively reported scores after the clinical outcomes of the patients treated on their 'shifts' were known [28] or after senior nurses had retrospectively abstracted subjective and objective data in patients with events [29]. This potential reporting bias might have inflated score performance. Finally, none of the trigger identification methods has been validated, although each has been used in before-and-after studies of rapid response team implementation [1,30,31].

### Limitations

There are four main limitations of this study. First, the absolute delineation of 'sick' and 'well' hospitalised children is challenging. The categorisation of children into clinical groups reflected a pragmatic decision. Dichotomisation is useful for score validation and may simplify clinical decision making, but it does not reflect the complex environment and clinical decision making in hospital inpatient units. Our definition of 'well' did not exclude children with complex clinical presentations, who may have been at significant ongoing risk for adverse outcomes, and other 'stable children' with consistently abnormal vital signs. Inclusion of these children increases the generalisability of our results and reflects the challenges of clinical decision making. These children provide the rationale for developing objective measures of the severity of illness, such as the Bedside PEWS score. Furthermore, the classification of a child as 'sick' on the basis of urgent ICU admission or a code blue call has limitations. The severity of illness in the first hours after ICU admission varies [32,33], and the decision to place an immediate call to a resuscitation team is complex, subjective and multifactorial [34].

Second, we relied upon observed data rather than specifying the frequency and nature of clinical observations. The frontline staff who cared for the children studied were unaware of the Bedside PEWS score and its component items and thus would not prospectively have known that their patients were being studied. Ideally, we would have prospectively obtained complete and identical clinical data from case and control patients; however, this was not possible, given the ethical and logistical challenges of identifying case patients in advance. The patterns of missing data may differ between case and control patients and thus may have influenced the calculated scores. Of the 23,288 hours studied, only 5.1% had measurements on all 7 items, indicating that incomplete data were very common.

Third, the patients for whom an immediate call was made to resuscitation teams may have been systematically different from other patients. These children may have had either rapid progression of their illness or underappreciation of an already concerning severity of illness, or both. These patients are the most challenging to identify prospectively. The lower scores found in patients who had a code blue event may reflect differences in patient monitoring or provider expectations. Prospective scoring of all patients using a standardised approach is required to resolve this question. Retrospective observational studies and studies of early intervention suggest that these adverse outcomes, including in-hospital cardiopulmonary arrest, are preventable [16,35-40]. Evaluation of the clinical impact of the implementation of the Bedside PEWS score is required to assess this potential.

Fourth, following abstraction, the Bedside PEWS score was calculated electronically after data collection without knowledge of the frontline nurse or the research nurse collecting the data. Consequently, we could not assess the accuracy or reliability of score calculation. This requires evaluation in future studies.

## Conclusions

We performed a multicentre case-control study to validate the Bedside PEWS score. In our evaluation of 2,074 patients, we found that, with at least one hour's notice, the Bedside PEWS score could distinguish 'sick' from 'well' hospitalised patients and that this score increased during the time leading up to events and was consistently high in case patients independently of the number of risk factors for near and actual cardiopulmonary arrest. Together these data suggest that the Bedside PEWS score can help clinicians to identify children at risk for near and actual cardiopulmonary arrest. Further evaluation of the clinical impact of the implementation of the Bedside PEWS score is required to assess its potential.

## Key messages

• Evaluation of clinical data from 2,074 patients in four paediatric hospitals showed that the Bedside PEWS score could identify children at risk of cardiac arrest with at least one hour's notice.

• After inclusion of the data from the hour immediately before near or actual cardiopulmonary arrest events, the AUCROC (95% CI) curve increased from 0.87 (0.85 to 0.89) to 0.88 (0.87 to 0.90).

• Bedside PEWS reflected evolving critical illness. Scores increased over the 24 hours before near or actual cardiopulmonary arrest events.

• The retrospective opinion of nurses caring for the patients studied was inferior to the Bedside PEWS score (*P *< 0.0001).

• Evaluation of the effect of the Bedside PEWS score on important clinical outcomes is required.

## Abbreviations

AUCROC: area under the receiver operating characteristic curve; Bedside PEWS: Bedside Paediatric Early Warning System.

## Competing interests

CSP and KM are the named inventors of the Bedside Paediatric Early Warning System. US and European patents are pending. As of April 2011, CSP and KM owned stock in Bedside Clinical Systems, a Clinical Decision Support company. The activities of this company include development of an electronic form of the Bedside Paediatric Early Warning System, of which the Bedside PEWS score is a component.

## Authors' contributions

CSP conceived of the study, contributed to its design, oversaw data acquisition, contributed to data analysis, wrote the initial draft of the manuscript and contributed to subsequent manuscript revisions. HPD, ARJ, CAF, JRL, KLM and JSH each contributed to the design of the study, contributed to data acquisition at their respective hospitals and contributed to manuscript revisions. PCP and DW contributed to the study design and manuscript revisions. JB contributed to the study design and analysis. NB contributed to study analysis and manuscript revisions. All authors read and approved the final manuscript.
